# Analyzing handwriting legibility through hand kinematics

**DOI:** 10.3389/frai.2025.1426455

**Published:** 2025-03-26

**Authors:** Vahan Babushkin, Haneen Alsuradi, Muhamed Osman Al-Khalil, Mohamad Eid

**Affiliations:** ^1^Applied Interactive Multimedia Lab, Engineering Division, New York University Abu Dhabi, Abu Dhabi, United Arab Emirates; ^2^Tandon School of Engineering, New York University, New York, NY, United States; ^3^Arabic Studies Program, New York University Abu Dhabi, Abu Dhabi, United Arab Emirates

**Keywords:** handwriting, deep learning, temporal convolutional networks, sensorimotor learning, machine learning

## Abstract

**Introduction:**

Handwriting is a complex skill that requires coordination between human motor system, sensory perception, cognitive processing, memory retrieval, and linguistic proficiency. Various aspects of hand and stylus kinematics can affect the legibility of a handwritten text. Assessing handwriting legibility is challenging due to variations in experts' cultural and academic backgrounds, which introduce subjectivity biases in evaluations.

**Methods:**

In this paper, we utilize a deep-learning model to analyze kinematic features influencing the legibility of handwriting based on temporal convolutional networks (TCN). Fifty subjects are recruited to complete a 26-word paragraph handwriting task, designed to include all possible orthographic combinations of Arabic characters, during which the hand and stylus movements are recorded. A total of 117 different spatiotemporal features are recorded, and the data collected are used to train the model. Shapley values are used to determine the important hand and stylus kinematics features toward evaluating legibility. Three experts are recruited to label the produced text into different legibility scores. Statistical analysis of the top 6 features is conducted to investigate the differences between features associated with high and low legibility scores.

**Results:**

Although the model trained on stylus kinematics features demonstrates relatively high accuracy (around 76%), where the number of legibility classes can vary between 7 and 8 depending on the expert, the addition of hand kinematics features significantly increases the model accuracy by approximately 10%. Explainability analysis revealed that pressure variability, pen slant (altitude, azimuth), and hand speed components are the most prominent for evaluating legibility across the three experts.

**Discussion:**

The model learns meaningful stylus and hand kinematics features associated with the legibility of handwriting. The hand kinematics features are important for accurate assessment of handwriting legibility. The proposed approach can be used in handwriting learning tools for personalized handwriting skill acquisition as well as for pathology detection and rehabilitation.

## 1 Introduction

Handwriting is a complex sensorimotor skill that requires simultaneous coordination between human visual-perceptual, cognitive, and motor systems (Bonney, 1992). Writers process visual and haptic feedback to coordinate the hand, arm and finger movement in order to produce legible handwriting. Developing legible handwriting is crucial for children development, and can affect the educational process, academic success, and self-confidence (Chang and Yu, 2013). Furthermore, understanding the factors that influence handwriting legibility is essential for various practical applications. These include designing personalized learning programs to cater to individual needs (Jenkins et al., 2016), identifying and addressing handwriting difficulties to support students' learning (Drotár and Dobeš, 2020; Fancher et al., 2018), and verifying individuals identities through handwriting analysis in security and forensic contexts (Galbally et al., 2007). Thus, it can be observed that the study of handwriting legibility can inform both educational strategies and technological applications, highlighting its broader significance.

Handwriting legibility is a characteristic of the handwritten text that contributes to its readability (Rosenblum et al., 2004). Handwriting legibility often relies on expert evaluation of the produced handwritten sample (van Drempt et al., 2011). Several global scales are used to evaluate the legibility of healthy adults' handwriting, particularly of medical personnel. For instance, a 4-points scale is utilized to classify the legibility of handwritten medical documents as “illegible”, “mostly illegible”, “mostly legible”, and “legible” (Rodríguez-Vera et al., 2002). Expert evaluation is based on analyzing visual features of the handwritten sample such as size, spacing, alignment, slant, and formation (Amundson and Weil, 1996; Feder and Majnemer, 2007; Fancher et al., 2018). Early studies identified five factors characterizing the legibility of handwriting, namely letter formation, spacing, alignment slant and quality of line (Freeman, 1915). Subsequent studies also suggest that letter formation, size, text alignment and spacing significantly influence the legibility of children's handwriting (Ziviani and Elkins, 1984; Graham et al., 2006). In a recent study, different machine learning approaches were utilized to evaluate the legibility and aesthetics of handwritten text from images of Bengali handwritten documents, reporting 85.74% and 86.69% F-score, for legibility and aesthetics evaluation, respectively (Adak et al., 2017).

Given handwriting is a dynamic process including kinematic, spatial, and temporal components; more objective and quantitative methods are developed based on these dynamic features (Rosenblum et al., 2003). A few studies used the stylus kinematics data and machine learning to evaluate the legibility of individual's signature. For instance, occidental signatures, that incorporate letters and signs into concatenated text with some flourishing elements, are considered (Galbally et al., 2007). To determine if the individual's name can be inferred from the signature, i.e., if signature is legible or illegible, five stylus kinematics features are recorded, including pen-tip coordinates, pressure, and slant, and utilized to engineer 20 global features characterizing the individual's signature. These features are used to train a Multilayer Perceptron (MLP) classifier, achieving 84.56 % accuracy for binary classification (Galbally et al., 2007). Other temporal features are also considered to evaluate the legibility of handwriting, including handwriting speed (Graham et al., 1998a), handwriting style (Graham et al., 1998b), the applied pressure (Harris and Rarick, 1959), and the grasping style (Schwellnus et al., 2012). The potential of these features to detect pathologies such as Alzheimer's disease (AD) from handwriting is explored in Wang et al. (2019), demonstrating that AD patients produce lower pen pressure and variations in the vertical direction, in comparison to healthy subjects.

While hand motion parameters such as fingers/palm position/orientation, acceleration/deceleration, and overall hand speed may influence legibility, they have yet to be explored. This study builds on the methods and findings outlined in PhD dissertation (Babushkin, 2024), and aims to examine correlates between hand/stylus kinematics and handwriting legibility. An experimental setup is developed to complete a handwriting task using a handwriting tablet while recording the stylus and the hand movement in 3D. A deep learning model, inspired by temporal convolutional networks (TCN), is constructed to evaluate the legibility of handwriting based on the time series kinematic data. We hypothesize that hand kinematic features play a prominent role in evaluating handwriting legibility. The interpretable machine learning approach (Shapley values) is used to identify prominent sensorimotor features derived from hand and stylus kinematics in evaluating the handwriting legibility.

## 2 Methodology

### 2.1 Experimental setup

**Figure 1:**
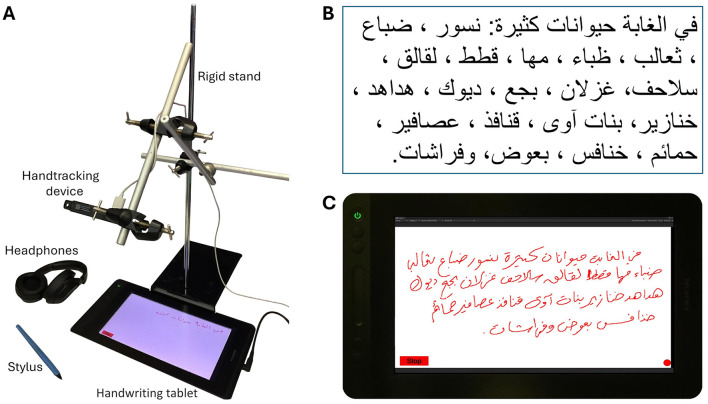
**(A)** Experimental setup, **(B)** sample text dictated to subjects, **(C)** the user interface, displaying a sample of Arabic handwriting recorded from a single subject (Babushkin et al., 2024).

The experimental setup (Figure 1) and protocol are based on a previously established methodology (Babushkin et al., 2024, 2023), with modifications to specifically investigate handwriting legibility. The experimental setup (Figure 1A) includes a HUION GT-116 tablet paired with a pen-like stylus and an Ultraleap Stereo IR 170 hand motion tracker. The hand tracking device, as shown in Figure 1A is attached to a rigid stand in a way that allows it to accurately track the writer's hand movements. The system can be easily moved, allowing for data collection in different locations.

### 2.2 Experimental task and protocol

Similarly to Babushkin et al. (2024), participants were instructed to write a text consisting of 26 Arabic words (Figure 1B), as this number was optimal for fitting within the dimensions of the recording tablet screen. The text was meticulously designed to cover all 28 letters of the Arabic alphabet and to include all key connectivity positions in Arabic orthography. Despite the unique glyphs (e.g., ك) were included in both their connected and unconnected forms, homoglyphs were not individually represented in all their connected/unconnected variations, but rather as a group (for example the ب, ت, ث homoglyphs).

The choice of Arabic script is justified by its cursive nature, context sensitivity, and multiple writing styles, which makes it more complex in comparison to the Latin script (Naz et al., 2013, 2014; Kacem et al., 2012). All these features of Arabic orthography allow to address a wider variety of handwriting skills.

The subject listened to the entire text sample at a speed of 20 words per minute before the start of the experiment. The experiment started when the participant pressed the “Start” button on the tablet screen to start recording of data (hand tracking and stylus), which turned red indicating recording was in progress and changed its label to “Stop”. The experimenter played the sample text to the subject from an audio recording, adjusting dictation speed according to the pace of the subject's handwriting. As soon as the dictation ended, the subject pressed the “Stop” button to submit the recording. The collected data contained tablet screenshots (Figure 1C), seven stylus-kinematic features recorded from the tablet, and 110 hand-kinematic features recorded by the hand tracking device. The handwriting task was repeated 6 times for each subject with the same text being dictated each time.

### 2.3 Participants

In total, 50 participants were recruited for this study. All participants were native Arabic speakers, above 18 years, who attended school with Arabic instruction language from grade 1 and with no previous history of neuromuscular or orthopedic dysfunction or dysgraphia. Additional inclusion criteria required participants to be available for in-person sessions to record handwriting tasks and to predominantly use their right hand for writing. The study was conducted in compliance with the Declaration of Helsinki, following its norms and regulations, and with an authorized protocol by the New York University Abu Dhabi Institutional Review Board (IRB: #HRPP-2023-93).

### 2.4 Expert evaluation and measures

Three Arabic teaching experts (all females, aged 35–55 years) were recruited to evaluate the legibility of the handwriting samples (image-based) using the eligibility evaluation form (see Supplementary Figure S1). To accommodate for the diversity in style of education systems, the experts represented three different educational and cultural backgrounds (Arabic gulf countries, North Africa, and Middle East). Furthermore, experts were recruited based on the following inclusion criteria: (1) having more than 10 years of experience in teaching Arabic handwriting, and (2) currently working in official (statutory work) or extra-official settings (non-statutory work).

**Figure 2:**
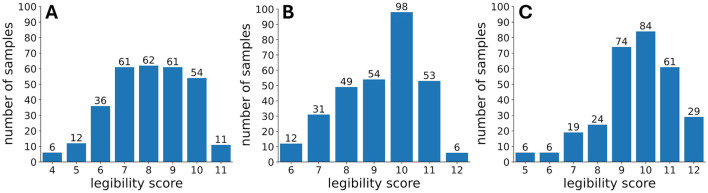
Cumulative score distributions by experts **(A)** expert 1, **(B)** expert 2, **(C)** expert 3.

The non-language dependent Handwriting Legibility Scale (HLS) (Barnett et al., 2018) was adapted to incorporate features specific to Arabic handwriting, such as aesthetics. The three Arabic teaching experts were tasked with evaluating the handwriting samples in terms of readability (how easy/difficult is it to read this person's handwriting?), space management (was this person able to fit their writing in the space available?), style consistency (how consistent was this person in following specific style?), and aesthetics (how beautiful was the handwriting?). Each of these questions was rated on a 3-point Likert scale, and a cumulative legibility score was calculated by summing the responses to these four questions, yielding a total score between 4 and 12 points. Furthermore, based on the experts' observations of high similarity among samples written by the same individual, the cumulative scores of all six samples from each subject were averaged within each subject. The resulting average score was then rounded to the nearest integer and assigned as the legibility score for all samples written by that subject. The cumulative legibility scores assigned by the three experts (see Figure 2) were nearly normally distributed, meaning that the handwriting legibility was well-represented within the recruited subjects.

In total 117 kinematic features were recorded from two different sources: seven stylus features from the tablet and 110 hand kinematics features from the hand tracking device (see Table 1). The data were collected at a sampling rate of 25 Hz and synchronized to a unified timestamp (Babushkin et al., 2024, 2023).

**Table 1:** The recorded 117 hand and stylus kinematics features (Babushkin et al., 2024, 2023).

**Modality**	**Features**
Stylus kinematics features	Stylus tip coordinates (*x, y, z*) (*z* = const), Pressure (applied force), Azimuth (angle of the stylus projection onto the tablet surface, counted clockwise), Altitude (angle between the tablet screen and the stylus), Proximity to the writing surface.
Hand kinematics features	• (*x, y, z*) coordinates of following Index, Middle, Ring and Pinky fingers' bones: • Distal, • Intermediate, • Proximal, • Metacarpal, • Proximal end of the metacarpal bone. (*x, y, z*) coordinates of following Thumb bones: • Distal, • Intermediate, • Proximal, • Metacarpal. Palm center (*x, y, z*) coordinates, Hand pinch position (*x, y, z*) coordinates (thumb and index if they are pinched), Hand predicted pinch position (*x, y, z*) coordinates, Hand wrist position (*x, y, z*) coordinates, Elbow position (*x, y, z*) coordinates (estimated if not in view), Hand arm center (*x, y, z*) coordinates (midpoint of the bone), Palm speed (*v*_*x*_, *v*_*y*_, *v*_*z*_) components, Hand palm normal (*n*_*x*_, *n*_*y*_, *n*_*z*_) coordinates, Hand rotational components (*r*_*x*_, *r*_*y*_, *r*_*z*_, *r*_*w*_), Palm width, Palm pitch, Palm yaw, Palm roll, Hand pinch strength, Hand pinch distance, Hand grab angle, Hand arm length (length of the bone), Hand arm width (average width of flesh around the bone).

### 2.5 Model architecture

The proposed model is motivated by the temporal convolutional network (TCN) (Lea et al., 2016) design that uses 1D convolutions to extract features encoded across time (Dai et al., 2019). Due to their ability to update layers' weights at every time step simultaneously, TCNs demonstrate better performance than Long-Short Term Memory networks while dealing with long time series (Zhang et al., 2017; Dai et al., 2019); they can take sequences of any lengths and ensure the absence of information leakage from future to past events (Yan et al., 2020). However, TCNs still face difficulties with inferring dependencies between long-range patterns due to the limited receptive field of the convolutional kernels (Dai et al., 2019). The addition of a self-attention layer to TCN enhances its ability to capture these long-range dependencies (Vaswani et al., 2017). Furthermore, the self-attention mechanism allows to infer hidden associations in features, enabling the network to learn irregular and complex patterns (Bu and Cho, 2020). Additionally, self-attention can also lead to more interpretable models (Vaswani et al., 2017).

**Figure 3:**
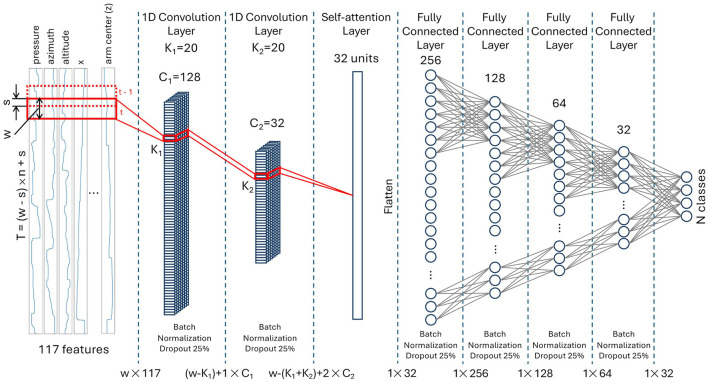
The model architecture with two temporal convolution layers. *T*, is the length of entire paragraph (can vary depending on the writing speed of the subject); *n*, number of windows; *w*, length of the window; *s*, overlap; *K*_1_, *K*_2_, kernel sizes of first/second 1D CNN layers; *C*_1_, *C*_2_, number of channels in first/second 1D CNN layers; *N*, number of classes.

The proposed model, illustrated in Figure 3, consists of two TCN layers represented by one-dimensional convolutional layers (1D CNN). These are followed by a self-attention layer that processes the hidden representation and extracts a global temporal attention mask. Learning takes place within the subsequent four fully-connected layers. Both the number of fully-connected layers and the number of neurons in each layer were determined empirically, starting with the simplest possible architecture.

The number of TCN layers used in the model is based on the assumption that the first layer captures temporal dependencies, while the second layer focuses on inferring spatial dependencies from the feature maps produced by the first layer. Visualization of feature maps after the first and second 1D CNN layers confirmed this assumption. Adding more than two 1D CNN layers leads to incremental improvements in the model's performance.

The input layer takes a matrix of 117 features and *t* time points and feeds it to the first convolution layer with number of channels *C*_1_ = 128. The original sample of length *T* = (*w* − *s*) × *n* + *s*, where *s* is overlap, is split into *n* windows of length *w*. The convolution is performed by sliding a kernel of size *K*_1_ = 20 along the time dimension of each window. The resulting (*w* − *K*_1_) + 1 × *C*_1_ matrix is passed to the input of the second 1D convolution layer of 32 channels with a kernel of size *K*_2_ = 20 sliding along the time dimension. The *w* − (*K*_1_ + *K*_2_) + 2 × *C*_2_ feature map matrix from the second 1D convolution layer is processed by self-attention layer of 32 units, then flattened and passed to the fully connected layers with 256, 128, 64, 32 and finally *N* neurons, where *N* is the number of classes. To stabilize the learning process and to prevent overfitting, the batch normalization and dropout of 25% were applied after convolutional and fully connected layers. The Rectified Linear Unit (ReLU) activation was used in all layers except the last one (output layer), which uses Softmax as an activation function. The model was trained on 200 epochs using categorical crossentropy loss function and Adaptive Moment Estimation (Adam) optimizer (Kingma and Ba, 2015). Due to the limited sample size, adjusting the learning rate using callbacks was not feasible – the optimal learning rate of 10^−3^ was found empirically and remained constant during the training. To combat the class imbalance, the oversampling method is applied to the training data, before using it to train the network.

### 2.6 Feature selection: Shapley values

Shapley values, initially introduced within game theory (Shapley, 1953), are used for assessing the influence of each feature on the prediction of the model (Lundberg and Lee, 2017; Fryer et al., 2021). The core concept behind Shapley values involves assessing the impact of each feature on the model's outcome by sequentially substituting each feature with uniformly-distributed random values and retraining the model on this new dataset. Shapley values are computed by comparing the model's predictions on the dataset with the random feature against the ones on the dataset with the original feature, for all instances in the validation set. These values are then averaged across the validation set to determine the overall influence of each feature. Ultimately, the distribution of averaged Shapley values is obtained, allowing to test the importance of the replaced feature compared to the substituted random feature.

The Shapley value of a feature of index *f* ∈ *F* = {1, …, *d*} from the set of all feature indices *F*, is a weighted average of all marginal contributions *M*_*f*_(*S*), each of them represents the difference in evaluation after introducing feature of index *f* to a sub-model *S* ⊂ *F*, i.e. *M*_*f*_(*S*) = *C*(*S* ∪ {*f*}) − *C*(*S*) (*C* is evaluation function). In this case, the Shapley value, ϕ_*f*_, of feature, *f*, is:


(1) $$\begin{array}{l} \phi_{f}=\underset{S\in 2^{F\backslash \left\{f\right\}}}{\sum }\omega \left(S\right)M_{f}\left(S\right), \end{array}$$

where $\omega \left(S\right)=\frac{|S|!\left(|F|-|S|-1\right)!}{\left|F\right|}!$ are the weights (Fryer et al., 2021; Babushkin et al., 2024, 2023).

### 2.7 Statistical analysis

Statistical analysis was conducted to understand how the most prominent features, extracted through Shapley values analysis, differed for samples with high and low legibility. The low and high legibility classes were selected for each expert following the legibility score distributions shown in Figure 2, i.e. for expert 1 the lowest legibility score was 4, highest was 11, for expert 2 lowest was 6, highest was 12, for expert 3 lowest was 5, highest was 12. The top 6 features, consistent across experts, namely pressure, azimuth, altitude and hand speed *x*, *y* and *z* components, were averaged over time for both the lowest and highest legibility classes. To ensure the independence of time-averaged features, the samples evaluated as high or low by more than one expert, were considered once only, i.e. there were no repetitions. The sample size for low legibility group for each feature was 619, and 1,384 for high legibility group. For each feature, the D'Agostino-Pearson omnibus normality test (recommended for large sample sizes D'Agostino and Stephens, 1986) was used to determine if the data follows normal distribution. The results demonstrated that the distributions for low and high legibility groups for all the 6 features were not normal and thus non-parametric Mann-Whitney U test was applied to evaluate statistical difference.

## 3 Results

### 3.1 Optimal parameter search

Legible handwriting is commonly evaluated based on the whole handwriting sample rather than a letter or a word (van Drempt et al., 2011). However, due to the limited sample size (303 paragraphs produced by 50 participants) and imbalanced distribution of the samples across the legibility classes, the sliding window method is adopted to enhance the sample size for model training and evaluation. Assuming the legibility of handwriting can vary within the text, the window should be large enough to contain sufficient number of words to ensure sufficient representation of the overall legibility score of the sample.

**Figure 4:**
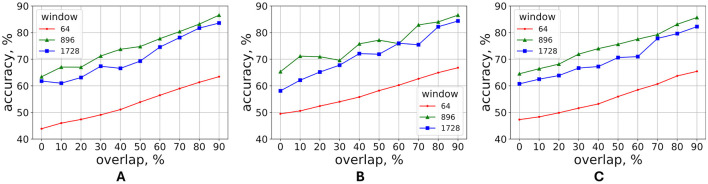
Parameters search—optimal overlap. For each expert, the model with optimal hyperparameters was evaluated for three different window sizes using 5-fold cross-validation across 10 evenly spaced overlap percentages values. The process was repeated five times, each with a different random seed. The accuracy values, averaged across five folds and five runs, for **(A)** expert 1, **(B)** expert 2, **(C)** expert 3.

To determine the optimal length of the time window, the grid-search technique was conducted for 27 different window lengths. Initially, the optimal overlap size was determined by training the model using three different fixed-length windows. Since the length of the window corresponding to the shortest sample was 1,774, the length of largest of these 3 fixed windows was selected as 1,728—a multiple of 64 which was close to 1,774. The smallest window length was set to 64 and the median window length was selected as 896. The overlaps were ranging from 0% to 90% with 10% step. While the number of words per given window of length *w* varied from subject to subject, the choice of step 64 was dictated by the minimum possible word length. The average accuracy was calculated over 5 folds and 5 runs for each expert and window length. The results indicated a linear increase in accuracy with the percentage of overlap (see Figure 4), leading to the adoption of a 90% overlap.

**Figure 5:**
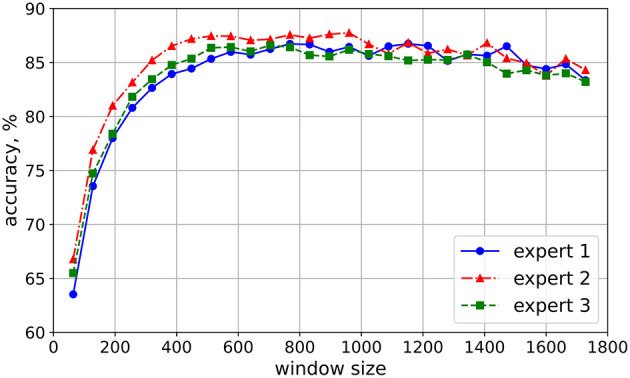
Parameters search—optimal window size. For each expert, the model with optimal hyperparameters was evaluated with 5-fold cross-validation across different window sizes, using 90% overlap. The evaluation was repeated five times, each with a different random seed. The accuracy values were averaged across five folds and five runs.

The parameter search was conducted to select a window containing sufficient number of words to justify the assignment of legibility score of the text sample to this window. The optimal window length was estimated by iterating over windows of lengths from 64 to 1728 with the step of 64 and overlap of 90%. For each iteration, the model was trained and validated for 5 folds. The process was repeated 5 times for each expert, each time with different random seed and the accuracy was averaged over all folds for each run. The results, presented in Figure 5, indicated that the model accuracy for each expert increased steadily from a window length of 64 to 576, plateauing around a window size of 1,408, and then declined slowly for larger windows. At window size of 704 all three experts were close to each other reporting an accuracy of around 84%. Therefore, a window size of 704 offered an optimal balance, achieving high accuracy while maintaining a minimized window length, which allowed for a larger number of samples.

### 3.2 Model performance evaluation

The proposed model was evaluated using 5-fold cross-validation in terms of accuracy, precision, recall and F1-score both with stylus alone and stylus and hand kinematics features. To avoid data leakage, the folds were formed at the paragraph level, before splitting a paragraph with sliding window of length 704 and 90% overlap. Thus all windows from the same paragraph were found only either in 4 folds for training or in the 5-th fold for testing, but not both in the training and testing folds simultaneously. The average performance of the model for each expert was summarized in Table 2. The confusion matrices are demonstrated in Supplementary Figure S2.

**Table 2:** Results of five-fold cross-validation for models using inputs from seven stylus kinematics features and 117 stylus and hand kinematics features for each expert.

	**Accuracy, %**		**Precision, %**		**Recall, %**		**F1-score, %**	
	**Stylus**	**Stylus and hand**	**Stylus**	**Stylus and hand**	**Stylus**	**Stylus and hand**	**Stylus**	**Stylus and hand**
Expert 1	76.0	86.3	78.8	88.4	74.8	89.3	75.8	88.6
Expert 2	76.5	87.2	76.4	86.9	73.2	87.0	73.7	86.4
Expert 3	75.3	85.4	75.8	88.4	73.8	84.5	73.4	85.8

The performance metrics were averaged over five folds.

Table 2 clearly demonstrates that the proposed model performed consistently well across all experts, with accuracy exceeding 85%. However, the model trained on expert 1 labels achieved the highest F1 score. It might be attributed to the distribution of expert 1 labels across classes being more uniformly distributed. On the other hand, expert 2 and expert 3 distributions were biased toward the high legibility scores, which suggests some leniency in evaluation. This leniency might be attributed to the educational system and cultural background of the last two experts.

### 3.3 Feature analysis

**Figure 6:**
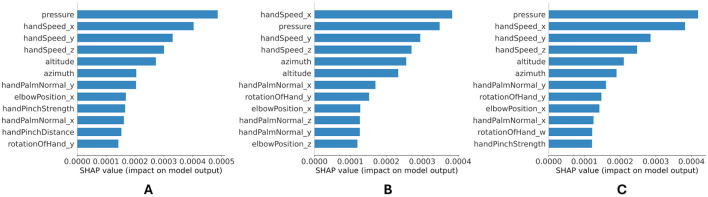
Aggregated Shapley values for each expert: **(A)** expert 1, **(B)** expert 2, **(C)** expert 3.

Shapley values were used to estimate the contribution of each feature in determining the legibility of handwriting. The three models, each trained for one expert's scores with optimal parameters were cross-validated for 5 folds. The Shapley values were evaluated for each fold with 500 samples from the testing set of the given fold. It was recommended to use the testing set to calculate Shapley values to better inspect the ML model and understand the model's decision-making process. However, Shapley values for the testing set allowed evaluating the features impact on the model's generalization performance. For each sample, Shapley values were calculated solely for the class corresponding to the true label of that sample. The obtained Shapley absolute values were averaged across 704 time-points and 500 test instances, and then aggregated for all 5 folds. The 12 most prominent features for predicting the handwriting legibility score by each expert are shown in Figure 6. It is clear that the pressure, hand speed components, and pen slant (altitude, azimuth) are consistently the top features across the three experts.

**Figure 7:**
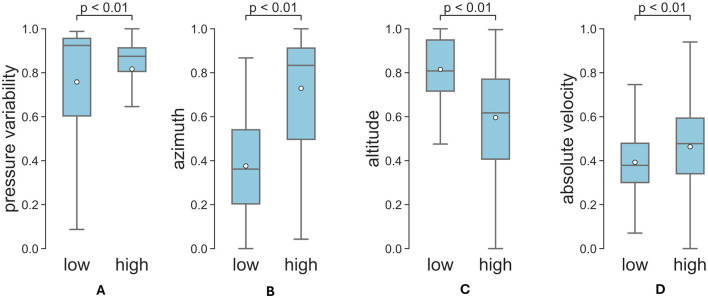
Top influential factors for low and high legibility, **(A)** pressure variability, **(B)** azimuth, **(C)** altitude, **(D)** hand absolute velocity. The scales were normalized to [0, 1] interval.

Figure 7 shows the results of the time-average of the top 4 features for low and high legibility. The average altitude is significantly higher for low legibility than high legibility (*p* < 0.01, Mann-Whitney U Test). On the contrary, azimuth, and hand absolute velocity $v=\sqrt{v_{x}^{2}+v_{y}^{2}+v_{z}^{2}}$, where *v*_*x*_, *v*_*y*_ and *v*_*z*_ are hand speed components, are significantly higher for high legibility as compared to low legibility (*p* < 0.01, Mann-Whitney U Test). According to Harris and Rarick (1959), the pressure itself does not necessarily correlate with legibility for healthy adults, but the pressure variability does. The pressure variability was also calculated as the standard deviation of pressure over time for each time window for high and low legibility classes. The Mann-Whitney U Test confirms that the pressure variability is significantly higher for low legibility (*p* < 0.01), which finds echo in previous literature (Harris and Rarick, 1959). Apparently, the good performance of the model is due to its ability to capture the differences between features from samples coming from different legibility groups.

Finally the correlation between stylus and hand kinematics features such as pressure variability and the absolute speed of handwriting was considered for writers whose handwriting samples (paragraphs) were evaluated as either highly legible or low legible. The paragraphs, produced by each one of those writers were unanimously evaluated either as highly legible or low legible by at least one expert. Highly legible paragraphs corresponded to the highest cumulative legibility scores assigned by the experts (11 for expert 1, 12 for experts 2 and 3; see Figure 2), while low legibility paragraphs corresponded to the lowest scores (4 for expert 1, 6 for expert 2, and 5 for expert 3; see Figure 2). The absolute velocities and pressure values were aggregated across all paragraphs for each subject. The mean absolute velocity and pressure variability were calculated from the aggregated data, resulting in 50 mean absolute velocity—pressure variability pairs, one for each subject. Both features were normalized to [0, 1] interval. The correlation coefficient between mean absolute velocity and pressure variability was 0.19, indicating a very weak relationship between these two features.

## 4 Discussion

The comparison of model performances with and without hand kinematics features (shown in Table 2) revealed the importance of including hand kinematics for more accurate evaluation of handwriting legibility. While the stylus kinematics features such as applied force, azimuth, and altitude might be sufficient for general legibility assessment, the inclusion of hand kinematics might detect subtle changes in legibility that can be used for diagnosing or predicting handwriting difficulties. Hand kinematics features, measured directly from hand tracking, capture hand dynamics more accurately than tablet features. While handwriting speed can be approximated from stylus kinematics features, it does not explicitly measure the hand speed, which is captured by a hand tracking device. Thus, the inclusion of the hand kinematics features provides the model with a more accurate analysis of the hand dynamics.

The increase in model performance with window overlap for each expert (Figure 4) can be viewed as a form of data augmentation. Given the limited number of samples, increasing the overlap percentage between consecutive windows boosts the training dataset size, thereby enhancing accuracy. Moreover, hand and stylus kinematics data are highly temporal, requiring consecutive samples for effective learning. Training with higher overlap enables the model to learn temporal dependencies. Additionally, the data augmentation helps mitigate overfitting by exposing the model to samples with slight variations in hand and stylus kinematics, ensuring better generalization across subjects.

The search for optimal window length revealed another interesting behavior of the model. Despite the fact that shorter windows provide larger number of samples, extremely short window lengths, that contain few to a fragment of a word, are not sufficient to make inference about handwriting legibility (see Figure 5) as smaller textual content within each window may not provide enough information for the model to accurately assess legibility. Consistent force patterns, which are crucial for assessing legibility, are observed at the sentence level rather than the word level (Harris and Rarick, 1957). Furthermore, since there is an established correlation between the variability of pressure and handwriting legibility (Harris and Rarick, 1959), the model apparently infers the variability of pressure from a longer time interval, equivalent to more than a few words. Similarly, for a human expert it might be hard to provide an accurate evaluation of the legibility of handwriting just by observing a few words. The slight drop in accuracy for larger windows can be explained by the decrease in training samples with the increase of window length as well as the drop in the ability of model to generalize given the increased information per window.

There are significant differences in hand and stylus kinematics features between low and high legibility. Specifically, the pressure variability (Figure 7A) is significantly higher for low legibility than for high legibility, which is also established in previous studies, despite the different approaches to measure the applied force (Harris and Rarick, 1959). Lower overall hand speed (Figure 7D) appears to be associated with low legibility. While previous studies only hinted that subjects who write faster receive lower legibility scores (Harris and Rarick, 1959), our analysis revealed the opposite effect. This finding can be influenced by the differences in how the hand speed is measured—in our study the whole hand is tracked, while in Harris and Rarick (1957) and Harris and Rarick (1959), the hand speed was inferred from the oscillographic records. Other factors that may have influenced this result include the use of electronic tablet (rather than a physical paper) and the lack of friction feedback on the tablet. Interestingly, our study found a very weak correlation between pressure variability and absolute handwriting velocity. This may be due to the fact that pen pressure and writing speed are governed by distinct motor control processes. In adults, pen pressure has been shown to positively correlate with activity in the wrist extensor and flexor muscles, whereas increased writing speed is associated with decreased activation of these distal muscle groups (Gerth and Festman, 2023; Naider-Steinhart and Katz-Leurer, 2007). Additionally, the weak correlation could be influenced by factors such as the non-linear relationship between velocity and pressure variability, as well as individual differences in writing styles among participants. Beyond speed and pressure variability, pen slant (altitude, azimuth) is also a significant factor influencing evaluation of handwriting legibility. Specifically, a smaller azimuth angle and larger altitude features are associated with low legibility. This finds echo in literature where a previous study found that the pen slant is associated with handwriting difficulty (Asselborn et al., 2020).

Despite the differences in experts' cultural and academic backgrounds, the explainability analysis conducted with Shapley values (Figure 6) suggests that the pressure variability, hand speed components, and stylus slant (altitude, azimuth) features are consistently important across experts. This means that in general, experts implicitly rely on these features to evaluate the handwriting legibility. Other features that vary across the experts, are expert-specific and signify the assessment style of each expert. The model explainability analysis can be used to identify features that are correlated with low legibility and suggest handwriting practices/interventions to target these features and improve more effectively the legibility of handwriting.

A few limitations should be acknowledged. First, the sample size of 50 subjects is considered relatively low. The majority of subjects (30) were aged between 18 and 25 years with males constituting only half of the female population. The analysis of kinematic features influencing the legibility of handwriting should be performed on a larger and more diverse sample size. Furthermore, the effect of some demographic parameters, such as age and gender, shall be investigated. In future studies more experts will be recruited to evaluate legibility of the handwriting samples for understanding how the cultural and educational background influences the human-based evaluation and using this knowledge to mitigate possible expert-related biases in cumulative legibility score. Another technology related limitation involves the intermittent hand tracking for some subjects, particularly for female participants mostly due to the hand shape and in some cases the use of sunscreen cream that caused difficulties for tracking with infrared camera. Future research should consider using other data acquisition systems that allow writing on a physical paper (e.g., Wacom Bamboo) or emulating a paper interaction (e.g., reMarkable).

## Data Availability

The raw data supporting the conclusions of this article will be made available by the authors, without undue reservation.

## References

[B1] Adak C. Chaudhuri B. B. Blumenstein M. (2017). “Legibility and aesthetic analysis of handwriting,” in 2017 14th IAPR International Conference on Document Analysis and Recognition (ICDAR) (Kyoto: IEEE), 175–182.

[B2] Amundson S. J. Weil M. (1996). Prewriting and handwriting skills. Occup. Ther. Child. 3, 524–541.

[B3] Asselborn T. Chapatte M. Dillenbourg P. (2020). Extending the spectrum of dysgraphia: A data driven strategy to estimate handwriting quality. Sci. Rep. 10:3140. 10.1038/s41598-020-60011-8 32081940 PMC7035284

[B4] Babushkin V. (2024). Analyzing Arabic handwriting through hand kinematics: A deep learning approach. Brooklyn, New York: New York University Tandon School of Engineering.

[B5] Babushkin V. Alsuradi H. Al-Khalil M. O. Eid M. (2024). Analyzing Arabic handwriting style through hand kinematics. Sensors 24:6357. 10.3390/s24196357 39409395 PMC11478569

[B6] Babushkin V. Alsuradi H. Jamil M. H. Al-Khalil M. O. Eid M. (2023). Assessing handwriting task difficulty levels through kinematic features: a deep-learning approach. Front. Robot. AI 10:1193388. 10.3389/frobt.2023.1193388 37779578 PMC10540189

[B7] Barnett A. L. Prunty M. Rosenblum S. (2018). Development of the handwriting legibility scale (HLS): A preliminary examination of reliability and validity. Res. Dev. Disabil. 72, 240–247. 10.1016/j.ridd.2017.11.013 29223112

[B8] Bonney M.-A. (1992). Understanding and assessing handwriting difficulty: perspectives from the literature. Aust. Occup. Ther. J. 39, 7–15. 10.1111/j.1440-1630.1992.tb01751.x

[B9] Bu S.-J. Cho S.-B. (2020). Time series forecasting with multi-headed attention-based deep learning for residential energy consumption. Energies 13:4722. 10.3390/en13184722

[B10] Chang S.-H. Yu N.-Y. (2013). Handwriting movement analyses comparing first and second graders with normal or dysgraphic characteristics. Res. Dev. Disabil. 34, 2433–2441. 10.1016/j.ridd.2013.02.028 23747934

[B11] D'Agostino R. B. Stephens M. (1986). Tests for Normal Distribution in Goodness-of-fit Techniques. New York: Marcel Decker.

[B12] Dai R. Minciullo L. Garattoni L. Francesca G. Bremond F. (2019). “Self-attention temporal convolutional network for long-term daily living activity detection,” in 2019 16th IEEE International Conference on Advanced Video and Signal Based Surveillance (AVSS) (Taipei: IEEE), 1–7.

[B13] Drotár P. Dobeš M. (2020). Dysgraphia detection through machine learning. Sci. Rep. 10:21541. 10.1038/s41598-020-78611-9 33299092 PMC7725992

[B14] Fancher L. A. Priestley-Hopkins D. A. Jeffries L. M. (2018). Handwriting acquisition and intervention: a systematic review. J. Occup. Ther. SchoolsEarly Intervent. 11, 454–473. 10.1080/19411243.2018.153463438257543

[B15] Feder K. P. Majnemer A. (2007). Handwriting development, competency, and intervention. Dev. Med. Child Neurol. 49, 312–317. 10.1111/j.1469-8749.2007.00312.x 17376144

[B16] Freeman F. N. (1915). An analytical scale for judging handwriting. Elem. Sch. J. 15, 432–441. 10.1086/454438

[B17] Fryer D. Strümke I. Nguyen H. (2021). Shapley values for feature selection: the good, the bad, and the axioms. IEEE Access 9, 144352–144360. 10.1109/ACCESS.2021.3119110

[B18] Galbally J. Fierrez J. Ortega-Garcia J. (2007). “Classification of handwritten signatures based on name legibility,” in Biometric Technology for Human Identification IV (Bellingham: SPIE), 64–72.

[B19] Gerth S. Festman J. (2023). Muscle activity during handwriting on a tablet: an electromyographic analysis of the writing process in children and adults. Children 10:748. 10.3390/children10040748 37189997 PMC10137273

[B20] Graham S. Berninger V. Weintraub N. Schafer W. (1998a). Development of handwriting speed and legibility in grades 1-9. J. Educ. Res. 92, 42–52. 10.1080/00220679809597574

[B21] Graham S. Struck M. Santoro J. Berninger V. W. (2006). Dimensions of good and poor handwriting legibility in first and second graders: Motor programs, visual-spatial arrangement, and letter formation parameter setting. Dev. Neuropsychol. 29, 43–60. 10.1207/s15326942dn2901_4 16390288

[B22] Graham S. Weintraub N. Berninger V. W. (1998b). The relationship between handwriting style and speed and legibility. J. Educ. Res. 91, 290–297. 10.1080/00220679809597556

[B23] Harris T. L. Rarick G. L. (1957). The problem of pressure in handwriting. J. Exp. Educ. 26, 151–178. 10.1080/00220973.1957.11010592

[B24] Harris T. L. Rarick G. L. (1959). The relationship between handwriting pressure and legibility of handwriting in children and adolescents. J. Exp. Educ. 28, 65–84. 10.1080/00220973.1959.11010642

[B25] Jenkins S. Williams M. Moyer J. George M. Foster E. (2016). “The shifting paradigm of teaching: Personalized learning according to teachers,” in Knowledge Works. Retrieved from: https://knowledgeworks.org/wp-content/uploads/2018/01/teacher-conditions.pdf (accessed March 14, 2025).

[B26] Kacem A. Aouïti N. Belaïd A. (2012). “Structural features extraction for handwritten arabic personal names recognition,” in Proceedings of the 2012 International Conference on Frontiers in Handwriting Recognition, ICFHR'12 (IEEE Computer Society), 268–273. 10.1109/ICFHR.2012.276

[B27] Kingma D. Ba J. (2015). “Adam: A method for stochastic optimization,” in International Conference on Learning Representations (ICLR) (San Diega, CA: ICLR).

[B28] Lea C. Vidal R. Reiter A. Hager G. D. (2016). “Temporal convolutional networks: A unified approach to action segmentation,” in Computer Vision-ECCV 2016 Workshops, eds. Hua G. Jégou H. (Cham: Springer International Publishing), 47–54.

[B29] Lundberg S. M. Lee S.-I. (2017). “A unified approach to interpreting model predictions,” in Advances in Neural Information Processing Systems 30, eds. Guyon I. Luxburg U. V. Bengio S. Wallach H. Fergus R. Vishwanathan S.. (New York: Curran Associates, Inc), 4765–4774.

[B30] Naider-Steinhart S. Katz-Leurer M. (2007). Analysis of proximal and distal muscle activity during handwriting tasks. Am. J. Occup. Ther. 61, 392–398. 10.5014/ajot.61.4.392 17685171

[B31] Naz S. Hayat K. Razzak M. I. Anwar M. W. Akbar H. (2013). “Arabic script based language character recognition: Nasta'liq vs naskh analysis”, in 2013 World Congress on Computer and Information Technology (WCCIT), 1–7. 10.1109/WCCIT.2013.6618740

[B32] Naz S. Razzak M. I. Hayat K. Anwar M. W. Khan S. Z. (2014). “Challenges in baseline detection of arabic script based languages,” in Intelligent Systems for Science and Information: Extended and Selected Results from the Science and Information Conference 2013 (Cham: Springer), 181–196.

[B33] Rodríguez-Vera F. J. Marin Y. Sanchez A. Borrachero C. Pujol E. (2002). Illegible handwriting in medical records. J. R. Soc. Med. 95, 545–546. 10.1258/jrsm.95.11.545 12411618 PMC1279250

[B34] Rosenblum S. Parush S. Weiss P. L. (2003). Computerized temporal handwriting characteristics of proficient and non-proficient handwriters. The American Journal of Occup. Therapy 57, 129–138. 10.5014/ajot.57.2.129 12674304

[B35] Rosenblum S. Weiss P. L. Parush S. (2004). Handwriting evaluation for developmental dysgraphia: process versus product. Read. Writ. 17, 433–458. 10.1023/B:READ.0000044596.91833.55

[B36] Schwellnus H. Carnahan H. Kushki A. Polatajko H. Missiuna C. Chau T. (2012). Effect of pencil grasp on the speed and legibility of handwriting in children. Am. J. Occup. Therapy 66, 718–726. 10.5014/ajot.2012.004515 23106992

[B37] Shapley L. (1953). A value for n-persons games. Ann. Mathem. Stud. 28, 307–318. 10.1515/9781400881970-018

[B38] van Drempt N. McCluskey A. Lannin N. A. (2011). A review of factors that influence adult handwriting performance. Aust. Occup. Ther. J. 58, 321–328. 10.1111/j.1440-1630.2011.00960.x 21957916

[B39] Vaswani A. Shazeer N. Parmar N. Uszkoreit J. Jones L. Gomez A. N.. (2017). “Attention is all you need,” in Proceedings of the 31st International Conference on Neural Information Processing Systems, NIPS'17 (Red Hook, NY: Curran Associates Inc.), 6000–6010.

[B40] Wang Z. Abazid M. Houmani N. Garcia-Salicetti S. Rigaud A.-S. (2019). Online signature analysis for characterizing early stage Alzheimer's disease: a feasibility study. Entropy 21:e21100956. 10.3390/e21100956

[B41] Yan J. Mu L. Wang L. Ranjan R. Zomaya A. (2020). Temporal convolutional networks for the advance prediction of ENSO. Sci. Rep. 10:8055. 10.1038/s41598-020-65070-5 32415130 PMC7229218

[B42] Zhang K. Zuo W. Chen Y. Meng D. Zhang L. (2017). Beyond a gaussian denoiser: Residual learning of deep CNN for image denoising. Trans. Img. Proc. 26, 3142–3155. 10.1109/TIP.2017.2662206 28166495

[B43] Ziviani J. Elkins J. (1984). An evaluation of handwriting performance. Educ. Rev. 36, 249–261. 10.1080/0013191840360304

