# Supplementary material for: Analyzing handwriting legibility through hand kinematics
Source: Front Artif Intell. 2025 Mar 26;8:1426455. doi: 10.3389/frai.2025.1426455 (PMC11979204; doi:10.3389/frai.2025.1426455)
Supplement: Supplementary file 1 [file Data_Sheet_1.pdf]

Supplementary Material

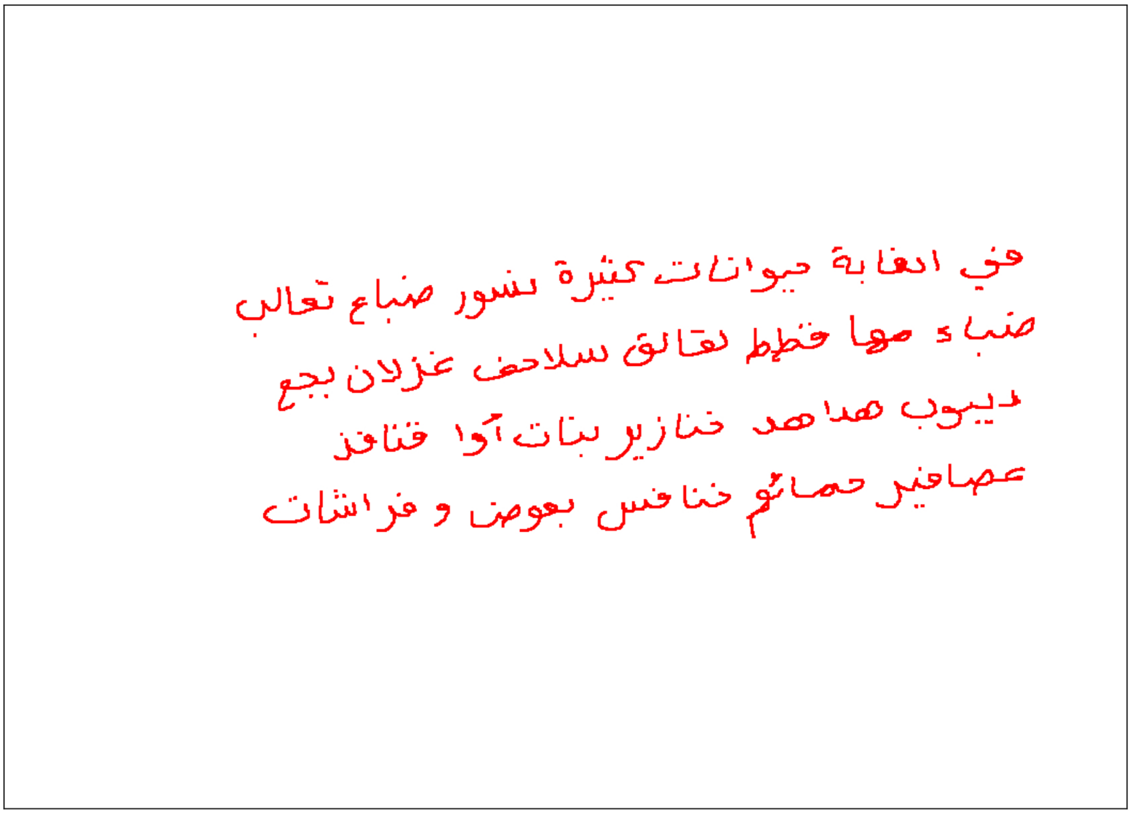

Word-level assessment

In the handwriting sample, please mark and count the following errors:

Spelling:

Letter formation:

Letter missing parts (hamza, tooth, etc.):

Dots:

Holistic assessment

In the following questions please consider your overall assessment of the writing:

Readability - How easy/difficult is it to read this person's handwriting?

☐ Hard to read ☐ Normal ☐ Easy to read

Space management - Was this person able to fit their writing in the space available?

☐ Not efficient ☐ Acceptable ☐ Efficient

Style consistency - How consistent was this person in following specific style (رقعة or نسخ or mix)?

☐ Not consistent ☐ Mixing ☐ Consistent

Aesthetics - How beautiful is the handwriting of this person?

☐ Not beautiful ☐ Normal ☐ Beautiful

Rate the overall legibility of this handwriting: ☐ Legible ☐ Illegible

Figure S1. Sample of the online paper-based survey filled by the experts. In the paper-based survey the experts were asked to perform the word-level assessment and evaluate their overall impression of the legibility of the given handwritten sample.

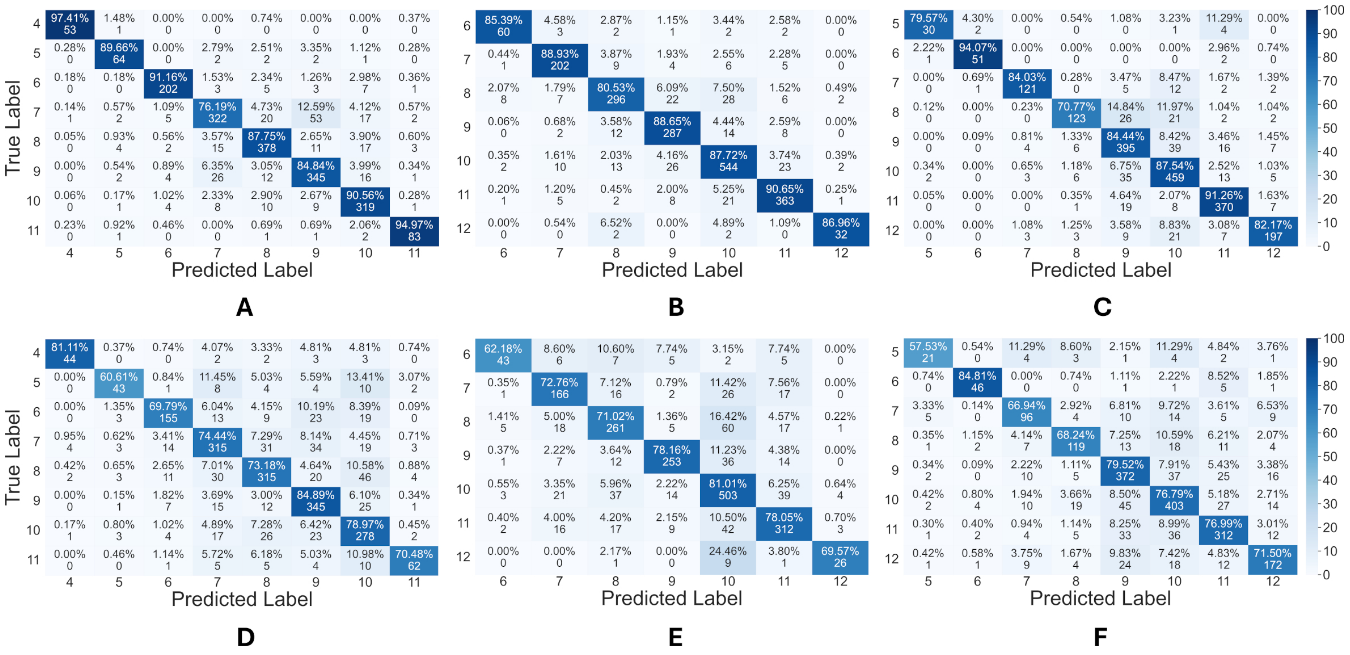

Figure S2. Confusion matrices for each expert averaged across 5 folds, upper row – model using stylus and hand kinematics features: (A) expert 1, (B) expert 2, (C) expert 3, lower row – model using stylus kinematic features only: (D) expert 1, (E) expert 2, (F) expert 3.
